# When there is no air, the cradle will fall: A narrative review of tobacco-related content across infant safe sleep interventions

**DOI:** 10.3389/fped.2022.994702

**Published:** 2022-12-05

**Authors:** Aysha Jawed, Mandeep Jassal

**Affiliations:** ^1^ Department of Pediatric and OB/GYN Social Work, Johns Hopkins Children’s Center, Baltimore, United States; ^2^ Department of Pediatrics, Johns Hopkins School of Medicine, Baltimore, United States

**Keywords:** sudden unexpected infant death, sudden infant death syndrome, infant safe sleep, tobacco, infant mortality, pediatrics

## Abstract

Sudden Unexpected Infant Death (SUID) from sleep-related causes is a leading cause of infant mortality worldwide. Sudden Infant Death Syndrome (SIDS) is one of the primary causes of SUID attributed to one or more environmental or behavioral determinants surrounding safe sleep practices among infants. The focus of many interventions on mitigating sleep-related infant deaths have addressed visible determinants pertaining to bed sharing, safe sleep surfaces, and removal of blankets, toys and other choking or strangulation hazards. Tobacco reduction and cessation have not been at the heart of any infant safe sleep interventions although addressing tobacco exposure is one of the primary safe sleep recommendations of the American Academy of Pediatrics. To date, there has not been a comprehensive review published on tobacco-related components across safe sleep interventions to reduce the risk of SIDS and SUID as the basis to contribute towards decreasing the rate of infant mortality. This review synthesizes the best practices, strategies, education, and additional interventions centered on addressing tobacco exposure as a risk factor for sleep-related infant deaths. Ten peer-reviewed studies were identified between 1995 and 2021 and integrated into this narrative review. There were three cross-sectional studies, three campaigns, one multi-center case control study, two randomized controlled trials, and two group comparison studies. Strengths and limitations of each approach are delineated followed by recommendations for future campaign, research, program, and practice endeavors to account for the totality of pertinent modifiable risk factors that contribute towards heightened infant mortality from sleep-related causes.

## Introduction

Sudden Unexpected Infant Death (SUID) is one of the leading causes of infant mortality across the world. Approximately, 3,400 infants under one year of age die annually from SUID in the United States (1). In fact, the vast majority of SUID cases are attributed to sleep-related causes. Furthermore, nearly 40% of SUID cases arise from Sudden Infant Death Syndrome (SIDS) (2). SIDS is characterized as the sudden unexpected death of an infant less than one year of age marked by the onset of the fatal episode resulting during sleep which remains unexplained after a thorough investigation inclusive of complete autopsy and review of the circumstances of death along with the clinical history (3). There are many environmental and behavioral determinants that can serve as both protective and risk factors for SIDS. Several of these determinants include sleep surfaces, environmental tobacco exposure, and heating conditions (4). All of them constitute environmental conditions in the Triple Risk Model which delineates the interface of infant vulnerability and critical time of development with environmental conditions in heightening an infant's susceptibility to SIDS (5).

The American Academy of Pediatrics (AAP) delineates recommendations for safe sleep practices as SIDS and SUID reduction measures among infants (Table 1). These recommendations include the following: supine sleep position, firm sleep surface, no bed-sharing, no soft objects and loose bedding, avoidance of smoke exposure, no overheating, breastfeeding, among many more (4). Each of these recommendations presents a target for intervention. Of note, the predictive and causal factors of SIDS and SUID are multifactorial. Focusing on one or a couple of these determinants (e.g., safe sleep surfaces) does not comprehensively account for the totality of pertinent risk factors that contribute towards the incidence and prevalence of infant mortality attributed to sleep-related deaths.

**Table 1:** American Academy of Pediatrics’ (AAP) 2022 safe sleep recommendations.

AAP Criteria	Level A Recommendations
A-1	Back to sleep for every sleep
A-2	Use a firm flat noninclined sleep surface
A-3	Room share without bed share
A-4	No soft objects and loose bedding
A-5	Prenatal care
A-6	Avoid smoke exposure
A-7	Avoid alcohol and other drugs (during pregnancy and after)
A-8	Breastfeed
A-9	Offer pacifier
A-10	Avoid overheating and head covering
A-11	Do not use home monitors
A-12	Immunize per schedule
A-13	Promote tummy time
A-14	Endorsement and modeling by healthcare providers of infant safe sleep guidelines from time of prenatal care
A-15	Media and manufacturers follow safe sleep guidelines in their messaging and advertising to promote safe sleep practices as the social norm
A-16	Continue to promote components of the Safe-to-Sleep campaign
**AAP Criteria**	**Level B Recommendations**
B-1	Avoid use of commercial devices that are inconsistent with safe sleep recommendations
**AAP Criteria**	**Level C Recommendations**
C-1	Avoid swaddling
C-2	Continue research and surveillance on risk factors, causes and pathophysiological mechanisms of sleep-related deaths

Many of these risk factors embody social determinants of health. The built environment, access to healthcare resources and services, socioeconomic status, and many more social determinants are inter-related and can impact health outcomes in either positive or adverse directions. Cultural factors also could represent either risk or protective factors. Both tobacco use and bed-sharing are significantly more prevalent in Eastern cultures, thereby heightening risk of sleep-related infant deaths across these communities (6).

Tobacco exposure is one of the leading forms of environmental smoke exposure that significantly increases the risk of SIDS and SUID among infants. From a postnatal perspective, environmental smoke exposure can impact the growth and immune system of infants given their smaller skin body composition (fat, muscle, and bone proportions) and compromise their airway which can further create respiratory complications that could be life-threatening, especially during time of sleep (7, 8). Prematurity and low birth weight attributed to prenatal tobacco exposure also heighten the risk of SIDS among infants (9). Of note, prenatal tobacco exposure creates an increasingly hypoxic environment for the fetus which potentially has implications for reducing brainstem-mediated cardiorespiratory control and sleep arousal for neonates postnatally (10). Similarly, unsafe sleep surfaces that are not in line with the AAP safe sleep recommendations represent a risk factor for SIDS.

Most interventions to address infant safe sleep have focused on a cluster of SIDS-related risk factors. These interventions have primarily involved addressing the most visible environmental determinants related to safe sleep surfaces and furniture (11). In fact, not a single safe sleep intervention has ever addressed every single safe sleep recommendation outlined by the AAP. Furthermore, tobacco reduction or cessation has also never been at the heart of any of these interventions likely given that the provider (e.g., pediatrician, family practitioner, obstetrician) for the SUID reduction intervention may not always be the provider (e.g., internist, family practitioner) for the recipient (caregivers) of the intervention. In addition, part of the complexities surrounding this issue center on variations in tobacco control policies (e.g., smokefree legislation, tobacco cessation) around tobacco exposure among the pediatric population (12). Of note, tobacco cessation efforts have demonstrated success among caregivers of infants and children in several prior studies (13) which further yields promise in integrating relevant content into infant sleep safety interventions. However, it has been integrated in parts of some safe sleep interventions as the basis to account for more SIDS-related risk factors. Furthermore, each of these risk factors impacts one another. In fact, multiple risk factors for SIDS are cumulative in effect. For example, infants sleeping in prone positions amidst shared bed spaces are more likely to be at a higher risk for SIDS with increased tobacco exposure (8).

It is crucial to account for both caregiver tobacco use and passive tobacco exposure given that they both contribute significantly towards increasing the risk of sleep-related infant deaths. Furthermore, both constitute the leading cause of environmental exposure among children and are inclusive of both traditional and electronic nicotine delivery systems (ENDS). In light of the growing vaping epidemic in recent years, the use of ENDS products across sociodemographics has continued to rise (14). Of note to date, there is limited knowledge on the long-term effects of the chemicals found in e-cigarettes, vape pens, and additional ENDS products which in turn heightens risk on the unknown health consequences of vaping and e-cigarette use and exposure on infants both pre and post-birth.

Infants are at a nearly three-fold increased risk of dying from SIDS if their mother smokes (15, 16). Furthermore, nearly two-thirds of SIDS-related deaths among infants could be prevented without both maternal smoking during pregnancy and passive tobacco exposure post-birth (15, 17–19). Addressing tobacco use and exposure prenatally continues to be a standard of practice; however, it is imperative to provide tobacco reduction and cessation support to caregivers on a continuum since risk of relapse increases significantly postnatally (20). In addition, it is crucial to account for caregivers from all walks of life that are or will be involved in the care of the infant both prenatally and postnatally inclusive of pregnant women, adoptive parents, and caregivers who identify across different gender groups.

To date, a comprehensive review of the literature has not been conducted to identify tobacco-related components of interventions to promote safe sleep practices as a predictor of reducing infant mortality attributed to sleep-related causes. The goals of this review are the following: (1) critically examine the best practices, strategies, education, messaging, and other interventions to address tobacco use in promoting safe sleep practices among infants; (2) assess outcomes that results from these clinical and organizational efforts; (3) reflect on strengths and limitations of each tobacco-related component across interventions; and (4) propose future directions in research and practice to target tobacco use as a modifiable risk factor for SIDS and SUID as the basis to contribute towards decreasing the risk of infant mortality.

## Materials and methods

### Search strategy

A narrative review of peer-reviewed literature on strategies, best practices, education and additional interventions on promoting safe sleep practices to reduce the risk of SIDS in infants across a range of community and healthcare settings was conducted in April 2022. The medical, public health, and psychosocial databases reviewed were the following: Medline, APA PsychInfo, Cochrane Review, Academic Search Premier, CINAHL, ERIC, and EBSCO. Key terms used across searches were the following: variants of sudden infant death syndrome, tobacco, smoking, vaping, neonate/infant/newborn/baby, pediatric, caregiver, parent, guardian, intervention, promotion, education, strategy, and best practices.

### Eligibility criteria

Peer-reviewed journal articles were included that involved practices, interventions, and strategies to address tobacco use and exposure as a risk factor for sleep-related deaths as the basis to and promote safe sleep practices among infants. Any articles that did not include tobacco-related components in the implementation of SIDS and SUID reduction interventions were excluded from this review.

### Procedure

Two authors independently reviewed all titles and abstracts across each selected database. Any differences concerning full-text inclusion were resolved through consensus. The authors then independently abstracted data across all included studies on best practices, strategies, education, and any other intervention characteristics, tobacco-related findings, and additional descriptive and qualitative information on the nature and implementation of interventions. Findings were subsequently compared and discrepancies were resolved through active discussions amongst the authors.

### Ethics

Institutional review board approval was not required for this narrative review.

## Results

**Figure 1:**
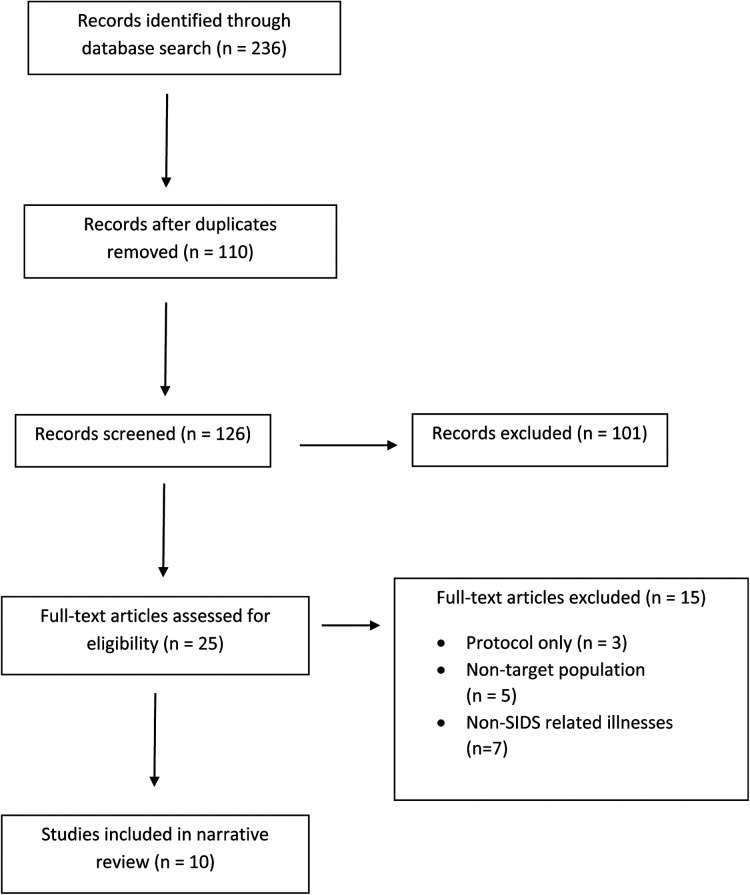
Narrative review flowchart.

A cumulative total of 236 records were identified across the databases reviewed from the past 26 years. 110 of these records were duplicates and ultimately excluded. Among the remaining 126 records, 101 of them were subsequently excluded for one or more of the following reasons: (1) did not contain full-text articles; (2) intervention components did not involve addressing tobacco dependence; and (3) did not implement interventions. 25 remaining full-text articles were examined for inclusion in this narrative review. Fifteen of them were ultimately further excluded for the following reasons: (1) non-target population; (2) presented only a study protocol; and (3) focused on a range of pediatric illnesses outside of SIDS. Ten of them ultimately met the criteria for presenting best practices, strategies, education, and any other interventions to address tobacco use as a risk factor for SIDS as elucidated in Figure 1. Ethics or IRB approval was obtained in seven studies (21–27). A consolidated breakdown of each study's design, intervention components, and outcomes can be found in Table 2.

**Table 2:** Study characteristics, tobacco-related components and findings.

First author, year, reference, *Location*	Trial Design	Participant Characteristics (Race/ethnicity, average age, % of tobacco users, sample size *N*, level of education)	Tobacco-Related Intervention Components	Tobacco-Related Findings
Ball, 2021 (21), *United Kingdom*	Cross-sectional, feasibility	97% Caucasian 21.4 years 35% smokers *N* = 79	Local midwives and health visitors wrote scripts and created videos for caregivers across health districts to emphasize local needs and services for smoking cessation, car seat safety, and maternal mental health, parents were provided with a leaflet on infant sleep safety that included content pertaining to breastfeeding, urging a smoke-free environment, and encouraging the back-sleep position (three major protective factors for SIDS), utilization of the infant sleep boxes among caregivers with tobacco dependence was strongly encouraged to reduce the risk of SIDS from a combination of co-sleeping with smoking, polypropylene box safety stickers were created with messaging about not letting anyone smoke around the baby	None reported
Hutton, 2017 (22), *United States*	RCT, feasibility	47% Black, 47% White, 5% biracial, 0.91% Asian, and 0.91% American Indian Intervention group: 22.14 years Control group: 21.89 years Intervention group: *N* = 160 Control group: *N* = 122 37% of participants had completed high school or earned their GED, 34% had completed some college level education, 25% had completed less than a high school education, and 1% were college graduates	Home visiting program for patients and their families receiving care through the Cincinnati Children’s healthcare system, received a brochure with messages promoting nonsmoking	None reported
McIntosh, 2018 (23), *New Zealand*	RCT	43.3% of participants each in the intervention and control groups were Maiori, approximately 40% of participants in each of the intervention and control groups were Pacific Intervention group: 13.33% under 20 years, 26.67% btwn 20-25 years, 43.33% > 26 years Control group: 13.33% under 20 years, 27.5% btwn 20-25 years, 41.67% > 26 years Maternal smoking a little greater than 50% in prevalence among pregnant women and less than 50% among women in post-partum	Development and implementation of community program	5% decrease in maternal smoking and smoke exposure four months post-intervention
Moon, 2004 (24), *United States*	Cross-sectional	Average age: 26.2 years *N* = 310 Level of education: 42.6% were high school graduates, 24.8% had acquired some college or technical school experience, 22.3% had not finished high school, 3.9% completed 4-year college, 2.9% were technical school graduates, 2.3% had completed postgraduate training, and lastly 1.3% had an unknown level of education 56.4% of households did not have any smokers while 30.9% of households had one smoker, 9.4% had 2 smokers, and 3.3% had at least 3 smokers	Educational intervention for caregivers of infants on safe sleep practices	None reported
Peacock, 2018 (25), *United States*	Qualitative	Not assessed given the qualitative content focus of the SIDS prevention campaign	Urban cities across the United States were assessed for the reach and efficacy of safe sleep public campaigns, dissemination of the following campaign materials: brochures, videos, rack cards, fact sheets, integrated nuanced risk reduction messages centered on bed-sharing with caregivers who are smokers and also emphasized that babies exposed to secondhand smoke are at an elevated risk of SIDS	None reported
Salm Ward, 2018 (26), *United States*	Group comparison	Average age: 29.04 years *N* = 132 92.8% of their participants as non-Hispanic with 90.1% of them as African Americans Level of education: nearly one-half of participants had completed either high school (35.6%) or some college level of education (28.8%)	Development and implementation of a safe sleep educational program across health departments in Georgia delivered by health educators, dissemination of cribs to all caregivers reached by the safe sleep educational program, messages centered on how smoke exposure increases risk of SIDS	None reported
Rivarola, 2016 (27), *Argentina*	Cross-sectional, group comparison	Average Age Intervention group: 26.98 years Control group: 26.59 years Intervention group: *N* = 267 Control group: *N* = 283 Level of Education Intervention group: 46% of them had completed either secondary, tertiary or university level education, 37% had obtained incomplete secondary education, and 17% had completed primary education Control group: 43% of them had completed either secondary, tertiary or university level education, 33.5% had incomplete secondary education, and 23.5% had completed primary education Intervention group: 5% of maternal caregivers smoked during pregnancy, 27% of maternal caregivers lived with a tobacco user Control group: 8.5% of maternal caregivers smoked during pregnancy and nearly 27.1% of them lived with a tobacco user	Dissemination of crib cards, posters, printed materials, parents were provided with a leaflet on infant sleep safety that included content pertaining to breastfeeding, urging a smoke-free environment, and encouraging the back-sleep position (three major protective factors for SIDS), the primary message integrated a combination of promoting supine sleeping position, breastfeeding, and breathing clean air	None reported
Ahlers-Schmidt, 2021 (28), *United States*	Cross-sectional	In-person community baby showers: *N* = 145 Virtual baby showers: *N* = 74 Among community baby showers conducted in-person, 60.4% of participants were non-Hispanic White followed by 20.8% non-Hispanic Black, 10.4% Hispanic, and 8.3% comprising other racial/ethnic groups; among participants engaging in virtual baby showers in the same study, 69.9% of participants were non-Hispanic White followed by 13.5% non-Hispanic black, 12.2% Hispanic, and 5.4% consisting of other racial/ethnic groups Level of education Across community baby showers delivered in-person, 54.9% of pregnancy and post-partum women were high school graduates or had earned their GED, 16.0% had some high school education, 8.3% were graduates of 2-year community college, 10.4% were graduates of 4-year college, 6.3% had completed graduate school, and 4.2% had a different level of education; among pregnant and post-partum women who had engaged in virtual baby showers, 43.2% had completed either high school or earned their GED, 6.8% had completed some high school, 17.6% had graduated from a 2-year community college, 17.6% had graduated from a 4-year college, 9.5% had completed graduate school, and 5.4% had a different level of education	Certified safe sleep instructors facilitated both in-person and virtual baby showers to promote safe sleep practices, education on safe sleep, breastfeeding, and tobacco cessation / avoidance, in-person events were interactive with presentation, demonstration, and video components, educational videos, prerecorded presentations, real-time interactive education on a virtual platform, participants received portable cribs and wearable blankets; knowledge on reducing secondhand smoke exposure and local resources for tobacco cessation were components integrated into the community baby showers to promote infant sleep safety	No significant changes in readiness to quit were observed before and after the community baby showers; among pregnant and postpartum women who participated in community baby showers held in-person, there was a slight increase (6%) in creating plans for tobacco-free homes and vehicles; in a different group of pregnant and postpartum women who participated virtually in these community baby showers, there was an 8% increase in developing plans to promote both tobacco-free homes and vehicles; pregnant and postpartum women in both groups expressed confidence in avoiding secondhand smoke exposure
Hill, 2004 (29), *Norway*	SIDS prevention campaign	First year: *N* = 5,539 Second year: *N* = 4,143 Level of education: approximately 11.4% of maternal caregivers had 16 or more years of total education while 67.6% had 11 or fewer years of education	Campaigns was developed and implemented in Norway, campaign-related messages were also disseminated *via* newspapers, journals, and magazines	No achieved tobacco reduction or cessation outcomes
Davidson-Rada, 1995 (30), *New Zealand*	Multi-center case control study	Not assessed given the nature of the SIDS prevention campaign	Campaign-related messages were disseminated *via* newspapers, journals, and magazines, parents were provided with a leaflet on infant sleep safety that included content pertaining to breastfeeding, urging a smoke-free environment, and encouraging the back-sleep position (three major protective factors for SIDS), messages centered on creating a smoke-free environment were delivered to parents along with messages to not smoke which were further reinforced by nursing to parents	None reported

### Sample characteristics

Sample sizes across studies ranged from 74 to 500 (21–24, 26–28). Ages of participants among studies ranged from less than 20 years to greater than 40 years (21–24, 26, 27, 29).

### Deliverers of interventions

In one study, certified safe sleep instructors facilitated both in-person and virtual baby showers to promote safe sleep practices (28). In another study, local midwives and health visitors wrote scripts and created videos for caregivers across health districts to emphasize local needs and services for smoking cessation, car seat safety, and maternal mental health (21). Health educators in two studies facilitated an educational intervention for caregivers of infants on safe sleep practices (24, 26). In another study, pediatricians, neonatologists, obstetricians, residents, licensed midwives, and nurses provided information to families of infants on safe sleep practices (27).

### Settings

In one study, baby showers were held both virtually and in-person across four rural counties (28). Another study recruited participants from two regions in the United Kingdom (21). In two different studies, campaigns were developed and implemented internationally in New Zealand and Norway (29, 30). Urban cities across the United States were assessed for the reach and efficacy of safe sleep public campaigns in a different study (25). Another study was also conducted in New Zealand through the development and implementation of a community-based program (23). One study involved implementing a home visiting program for patients and their families receiving care through the Cincinnati Children's healthcare system (22). Another study involved development and implementation of a safe sleep educational program across health departments in Georgia (26). A different study involved recruiting caregivers of infants in a WIC clinic at the Children's National Medical Center (24). Lastly, one study was implemented at maternity centers in Argentina (27).

### Study designs

Study designs were diverse across studies and included the following: cross-sectional to assess feasibility (21, 22, 24, 27, 28), SIDS prevention campaigns (25, 29, 30), multi-center case control study (30), randomized controlled trial (22, 23), and group comparison (26, 27).

### Data analyses

Data analyses across studies were primarily quantitative in nature and included the following: descriptive statistics, frequencies and percentages, McNemar tests across paired dichotomous variables, Friedman test, chi-square, paired sample t-tests, the Mann-Whitney Wilcoxon test for independent samples, Shapiro-Wilk test, linear mixed effects model, quantile-quantile plot, ratios of risk factor prevalence with stratified contingency table analysis, and logistic regression analysis (22, 23, 26–29). These analyses assessed descriptive contexts of variables as well as among pairs and groups.

One study involved conducting qualitative analyses through assessing the content of messages across campaign materials and interviews (25).

Quantitative data analyses were conducted using both SPSS (26, 28, 29) and SAS (21). Qualitative data analyses were performed with Atlas (25).

### Methods of health communication

In five studies, participants were reached *via* messages delivered through a range of diverse mass media methods that included social media, radio advertisements, posters, billboards, printed materials (e.g., pamphlets, leaflets, stickers) and fliers (22, 25–28). In addition, participants in two studies were recruited in the community by healthcare providers as well as maternal and child health programs and health departments (28, 30). Campaign-related messages were also disseminated *via* newspapers, journals, and magazines in two studies (29, 30).

### Provision of resources

Participants in one study received portable cribs and blankets (28). In another study, caregivers received infant sleep boxes made of polypropylene with wearable fleece baby blankets and fitted cotton sheets (21). One study involved the dissemination of cribs to all caregivers reached by the safe sleep educational program (26). In three studies, parents were provided with a leaflet on infant sleep safety that included content pertaining to breastfeeding, urging a smoke-free environment, and encouraging the back-sleep position (three major protective factors for SIDS) (21, 27, 30). In another study, the control group received a brochure with messages promoting nonsmoking (22). Baby beds were provided to participants in a different study (23).

### Education

In one study, knowledge on reducing secondhand smoke exposure and local resources for tobacco cessation were components integrated into the community baby showers to promote infant sleep safety (28). In another study, utilization of the infant sleep boxes among caregivers with tobacco dependence was strongly encouraged to reduce the risk of SIDS from a combination of bed-sleeping with smoking (21). Also in this study, polypropylene box safety stickers were created with messaging about not letting anyone smoke around the baby (21). Of note, this program was conducted in the United Kingdom where there is less emphasis on discouraging bed-sharing than in the U.S. In a different study, messages centered on creating a smoke-free environment were delivered to parents along with messages to not smoke which were further reinforced by nurses to parents (30). Two campaigns in Houston, TX and Washington DC integrated nuanced risk reduction messages centered on bed-sharing with caregivers who are smokers and also emphasized that babies exposed to secondhand smoke are at an elevated risk of SIDS (25). Similarly in another study, messages centered on how smoke exposure increases risk of SIDS (26). These messages on mediating secondhand smoke exposure to promote a smoke-free and vape-free environment resonate with messages from the Safe-to-Sleep and Cribs for Kids national campaigns (31, 32). Both of these campaigns promote adherence to the AAP infant sleep safety recommendations as the basis to optimize conditions for supporting an infant in sleeping safely and soundly without risk for any adverse consequences resulting from environmental and behavioral risk factors. Each of these campaigns has a visible presence across the U.S. and has existed for greater than twenty years (31, 32). In a different study, the primary message integrated a combination of promoting supine sleeping position, breastfeeding, and breathing clean air (27).

### Readiness to quit

In one study, no significant changes in readiness to quit were observed before and after the community baby showers (28).

### Environmental changes

In one study among pregnant and postpartum women who participated in community baby showers held in-person, there was a slight increase (6%) in creating plans for tobacco-free homes and vehicles (28). Of note in the same study in a different group of pregnant and postpartum women who participated virtually in these community baby showers, there was an 8% increase in developing plans to promote both tobacco-free homes and vehicles (28). Pregnant and postpartum women in both groups expressed confidence in avoiding secondhand smoke exposure.

### Tobacco cessation

There was no achieved tobacco reduction or cessation outcomes overall in one study that implemented a campaign across Norway (29). In fact, the prevalence of maternal smoking in the post-partum timeframe increased by approximately 3% following the campaign. Of note, among mothers who were 40 years of age and older in the same study, the prevalence of maternal smoking decreased by almost 5%. Furthermore, prevalence of maternal smoking was nearly 3.8 times higher among younger mothers than older mothers. In another study, there was a 5% decrease in maternal smoking and smoke exposure four months post-intervention (23).

## Discussion

We conducted a comprehensive narrative review of ten studies that described a range of different strategies, best practices, campaigns, and programs that accounted for tobacco exposure as a risk factor for SIDS and SUID to optimize safe sleep practices among caregivers of infants. However, none of the studies accounted for tobacco exposure as a primary focus of any intervention or an integral part of any SIDS reduction interventions. Content of tobacco-related messages across most of these studies was not clearly elucidated. Furthermore in the studies with tobacco-related messages, content centered on tobacco abstinence and/or reducing secondhand smoke exposure which are in line with content across both the Safe-to-Sleep and Cribs for Kids national campaigns to promote a tobacco-free environment for infants (31, 32). Studies only offered resources on safe sleep furniture, not any resources related to tobacco reduction or cessation among caregivers. Of note, only one study revealed tobacco-related outcomes pertaining to reduction or cessation. However, all studies integrated content on tobacco dependence and exposure as intervention components. An incomplete understanding exists of strategies to address more of the modifiable risk factors that can substantially elevate the risk of infant mortality from sleep-related deaths among infants. In our review, we have characterized pertinent components of each study design and key findings across studies. Furthermore, our review delineates both strengths and limitations of existing approaches as the basis to propose recommendations for future research and practice endeavors that can account for more environmental and behavioral determinants of SIDS and SUID inclusive of tobacco exposure.

### Limitations in resource provision

Of note, none of the studies involved provision of concrete tobacco cessation resources (e.g Nicotine Replacement Therapy products, referrals to tobacco treatment clinics, concurrent substance use and mental health treatment, etc.). The only concrete resources supplied across studies were cribs, boxes, pack-n-plays, and baby beds (21, 23, 26, 28). These resources address a couple of the AAP Safe Sleep recommendations for building a safe sleep environment for infants with firm sleep surfaces which seek to promote supine sleeping position and eliminate soft objects, loose bedding, and bed sharing. It is crucial for future interventions to also provide resources that can address more of the prescribed environmental recommendations inclusive of tobacco exposure.

### Delimitations of interventions

In addition across interventions, not only were resources supplied pertaining to safe sleep surfaces for infants, most interventions also heavily focused time and consideration on addressing unsafe sleep surfaces as a risk factor for SIDS and SUID. It is imperative to note that there are a range of significant preventable risk factors that can elevate an infant's risk of mortality from sleep-related causes that include tobacco exposure which can compromise the infant's airway and create subsequent complications in breathing that can be life-threatening.

Findings across these studies as well as through prior national infant safe sleep campaigns elucidate that tobacco reduction and cessation are not emphasized enough to reduce the risk of infant deaths postnatally attributed to behavioral and environmental determinants. Minimizing tobacco exposure is one of the guidelines published in 2022 by the American Academy of Pediatrics to optimize infant sleep safety. Oftentimes, postnatal care does not involve tobacco reduction or cessation care that was initiated prenatally. In turn, we are recommending that future work account for these outcomes.

In fact to date, there has not been a single intervention that has either exclusively or significantly focused on tobacco use as a modifiable risk factor for sleep-related infant deaths. Designing a comprehensive intervention that accounts for the complete range of behavioral and environmental determinants will likely mitigate the risk of infant mortality on a larger scale by addressing all potential preventable causes of them.

### Sources of environmental tobacco exposure

Of note, only two studies assessed whether there were any additional tobacco users at home (24, 27). It is important for future studies to identify all sources of environmental smoke exposure as the basis to mitigate them and thereby contribute towards the goal of SIDS and SUID reduction to subsequently decrease the rate of infant mortality.

### Children's literature

In one study among the intervention group, a children's book was published and include content on safe sleep recommendations (22). This book was narrative in nature and integrated a wealth of descriptive strategies on creating a healthy and safe sleep environment for infants. Of note, this book did not include any content on addressing smoke exposure as a threat to the safety of infants. The control group in this study received brochures with messages about not smoking. As previously mentioned, addressing tobacco exposure is one of the AAP's safe sleep recommendations. The fact that it was not accounted for suggests minimal importance of it and contributes to the knowledge gap in understanding and addressing the range of environmental determinants that surround the increased risk of SIDS and SUID. Children's books offer creative opportunities to engage caregivers of infants that include parents, grandparents, extended family members, child care providers, foster families and any other legal guardians of children. Future books could illustratively account for smoke exposure alongside additional safe sleep recommendations to engage this caregiver population and heighten their knowledge and awareness of more risk factors for sleep-related infant deaths.

### Tobacco control policies and legislation

Tobacco control measures have the potential to be effective in the form of legislation, taxation and social engineering. Findings from the World Health Organization's MPOWER report uncovered that 23 countries (Seychelles, Mauritius, Costa Rica, Brazil, Panama, Surinam, Colombia, Canada, Uruguay, Argentina, United Kingdom, Turkey, Portugal, Russia, Ireland, Romania, Estonia, Denmark, Spain, Norway, Iran, Australia and New Zealand) obtained the highest scores with respect to the implementation and enforcement of their tobacco control policies and legislation including smoke-free regulations, advertising bans, taxation, and uptake in the visibility of health warnings on cigarette packages (33). For example in Norway, smoking is entirely prohibited. Ever since the tobacco prohibition was implemented across restaurants, public transport, schools, healthcare institutions, as well as across all public office spaces in 2004, the prevalence of smoking in pregnancy has significantly decreased from 26% in 1999 to nearly 2% in 2021 (34). After the enactment of smoke-free legislation in Brazil, the average infant mortality rate declined substantially from 24.5 to 13.0 deaths per 1,000 live births from 2000 to 2016. During this time, the neonatal mortality in Brazil declined from 15.6 to 9.0 deaths per 1000 live births (35). It follows that integration of these tobacco control measures could also translate to addressing tobacco-related components of sleep-related infant deaths on a continuum from prenatal to postnatal care.

### Program expansion recommendations

There were several studies that involved development and implementation of community programs to address risk factors for SIDS and SUID as the basis to decrease preventable infant deaths (22, 23, 26). All of these interventions were implemented postnatally. Future endeavors could involve consideration of integrating tobacco dependence treatment in SIDS reduction approaches prenatally that could extend post-birth on a continuum for caregivers which could also heighten focus on tobacco-related outcomes both prenatally and postnatally. As previously mentioned, not a single study involved provision of tobacco cessation resources including medications, counseling, and referrals to tobacco treatment clinics. It is crucial for future programs to account for these resources as the basis to more comprehensively address more risk factors for infant mortality from sleep-related causes and follow as many AAP Safe Sleep recommendations as possible. Furthermore, accounting for cultural considerations, patient and family preferences, and varying degrees of health literacy can also reach and engage more subgroups within the target population of these diverse caregivers. Of note, cultural groups vary in their perspectives on what a safe sleep environment looks like (e.g., with respect to safe sleep furniture, severity of tobacco exposure, etc). Community programs can reach increasingly more caregivers who could benefit from these resources, inform delivery of care in line with the goals, values, and preferences of these caregivers, and in turn contribute towards decreasing the rate of infant mortality attributed to environmental and behavioral risk factors.

### Campaign-building recommendations

Several studies involved the development and implementation of campaigns to heighten knowledge and awareness of SIDS across a range of both traditional and nontraditional sources of media (25, 29, 30). It is unclear about the totality of messages delivered through this campaign and specifically with respect to tobacco-related messages as part of creating a safe sleep environment for infants. Among the messages shared in these studies, tobacco-related content centered on promoting smoke-free homes and vehicles and minimizing secondhand smoke exposure – both informational appeals similar in nature to the Safe-to-Sleep and Cribs for Kids national campaigns. Perhaps accounting for messages containing fear-based and emotional/psychological appeals in line with some of the well-known tobacco cessation campaigns (e.g., the CDC Tips from Former Smokers and the FDA's Every Try Counts and the Real Cost) along with tips and strategies to quit could be helpful in future campaigns to address tobacco exposure as a risk factor for SIDS (36–39). However before integrating any of these appeals into future endeavors, it is crucial to test each of them prior to implementation given the implication that this typology of intervention has the potential to be traumatic for caregivers. Furthermore, these campaigns have extended onto social media platforms that in turn increase the global reach for caregivers who comprise the primary target population for these campaigns. It follows that addressing more SIDS-related risk factors through the dissemination of messages across community, national, and global infant safe sleep campaigns could ultimately seek to reduce SIDS and SUID as part of the leading causes of infant mortality across the world.

### Limitations of this narrative review

This review's primary limitation is that we did not conduct a systematic review with meta-analyses. The narrative design of this review was more descriptive and qualitative in nature and in turn did not involve conducting composite statistical analyses. These limitations delimited rigorous examination of study biases. It follows that we could not critically assess whether any of the interventions, strategies, and practices pertaining to addressing tobacco exposure as a risk factor for SIDS could be directly related to health outcomes for infants and their caregivers. Lastly, we reviewed studies only published in English which could certainly be another limiting factor of this review.

## Conclusions

Existing interventions to mitigate sleep-related infant deaths do not account for more of the AAP Safe Sleep recommendations and delimit the reach and scope of their interventions with their focus on exclusive risk factors for SIDS and SUID. It is crucial for future interventions to account for the depth and breadth of more risk factors that are modifiable and could contribute towards the larger goal of reducing infant mortality overall by decreasing the risk of infant deaths attributed to sleep-related causes. More methods of health communication could focus content on emotional/psychological and fear-based appeals along with tips and strategies to promote tobacco cessation as a safe sleep recommendation alongside optimization of additional environmental and behavioral determinants constituting safe sleep practices. Children's books, programs, and campaigns offer unique opportunities to reach caregivers of infants and heighten their knowledge and awareness on SIDS and SUID reduction.
